# Prospects for automated diagnosis of verbal autopsies

**DOI:** 10.1186/1741-7015-12-18

**Published:** 2014-02-04

**Authors:** Michel Garenne

**Affiliations:** 1 Institut Pasteur, Epidémiologie des Maladies Emergentes, Paris, France; 2 IRD, UMI Résiliences, Bondy, France; 3 Witwatersrand University, School of Public Health, Johannesburg, South Africa

**Keywords:** Cause of death, Verbal autopsy, Automated diagnosis, Health information system, Evaluation of health programs, Public health

## Abstract

Verbal autopsy is a method for assessing probable causes of death from lay reporting of signs, symptoms and circumstances by family members or caregivers of a deceased person. Several methods of automated diagnoses of causes of death from standardized verbal autopsy questionnaires have been developed recently (Inter-VA, Tariff, Random Forest and King-Lu). Their performances have been assessed in a series of papers in *BMC Medicine*. Overall, and despite high specificity, the current strategies of automated computer diagnoses lead to relatively low sensitivity and positive predictive values, even for causes which are expected to be easily assessed by interview. Some methods have even abnormally low sensitivity for selected diseases of public health importance and could probably be improved. Ways to improve the current strategies are proposed: more detailed questionnaires; using more information on disease duration; stratifying for large groups of causes of death by age, sex and main category; using clusters of signs and symptoms rather than quantitative scores or ranking; separating indeterminate causes; imputing unknown cause with appropriate methods.

Please see related articles: http://www.biomedcentral.com/1741-7015/12/5; http://www.biomedcentral.com/1741-7015/12/19; http://www.biomedcentral.com/1741-7015/12/20; http://www.biomedcentral.com/1741-7015/12/21; http://www.biomedcentral.com/1741-7015/12/22; http://www.biomedcentral.com/1741-7015/12/23.

## Background

Interest in causes of death for public health purposes goes back to the 17^th^ century in London, when “death searchers” were recording deaths in the population by weekly household visits, with the main target being to estimate mortality from the plague. Since then the needs to have an accurate assessment of causes of premature deaths have only increased. Such needs are well covered in developed countries by a combination of routine compulsory death registration and medical diagnosis of each death. In many developing countries, however, death registration is still incomplete and causes of death remain largely undocumented because many deaths occur outside health facilities. Uncovering the reasons behind causes of death is important, especially in these settings, since better knowledge of the leading causes of death can help formulate policies to combat these and evaluate current strategies and health programs.

Verbal autopsies (VAs) were developed to bridge this gap. At first, they were conducted in research settings by an in-depth interview with the family of the deceased person. A good example is the Narangwal research project in India, where the term “verbal autopsy” was coined in the early 1970s [1]. This approach was limited by its cost and by the potential bias of a single observer. The next step was to use systematic questionnaires on a detailed history of the disease, signs, symptoms, treatments and any contextual information, including risk factors. This approach was less costly, more objective and allowed for some kind of proof for the final diagnosis. Several questionnaires were developed in the late 1970s and early 1980s for maternal deaths in Egypt [2], for neonatal and children deaths in Bangladesh [3], and for all causes in Senegal [4,5], which were further developed and adapted to a great variety of situations. They were used in research projects, in Demographic Surveillance System (DSS) sites such as Agincourt in South Africa [6-8], and soon were tried on representative samples of national populations as early as 1988 in Morocco [9-11], in a few Demographic and Health Surveys (Ghana 2007; Afghanistan 2010), and now on a very large scale in countries such as Mozambique, India and China.

The large scale application of verbal autopsies raises two issues: the quality and details of the questionnaire and the method of analyzing its content in order to reach a probable cause. Two types of methods for analyzing the questionnaire content are available: the first type is a judgment made by expert physicians and the second type is by use of an automated computer program. The first method can be relatively costly on large samples, may be slow if not enough human resources are available, and may not be fully replicable because of observer bias. Its main advantage lies in the ability of experts to make a judgment based not only on the strict reading of signs and symptoms but also on the local context, as well as on the feeling of whether the questionnaire has been properly filled out or not. The second method has the opposite advantages and disadvantages: low cost, fully replicable, but largely blind to the context and quality of information. Ideally, one would like a method both efficient and precise. The Niakhar project in Senegal, which was instrumental in developing verbal autopsy questionnaires, tried, in the mid-1980s, to develop automated diagnoses using a LISP (List Programing) artificial intelligence program. However, this project was stopped because, in order to supply the program with all the information necessary to make an automated diagnosis one had to complete a first analysis of the questionnaire and to recode numerous items, so that at that point the diagnosis was already made by hand. This did not mean that automated methods were not appropriate, but simply that they needed more preparatory work.

## New methods of automated diagnosis

A series of articles [12-16] in *BMC Medicine* has assessed some recently developed methods of automated diagnosis of questionnaire-based verbal autopsies. The main objectives of these automated methods are to use them on a large scale and to provide a profile of causes of death in a population for a variety of purposes, in particular, health information, evaluation of health programs and planning health interventions. Therefore, assessment of their performance is vital. The following automated methods were assessed:

1) “Inter-VA” was developed by Peter Byass and colleagues [17] and was improved over the years. The current version 4 is based on positive answers to a fixed list of 245 signs and criteria, from which a program computes the likelihood of a cause, and selects the cause with the highest likelihood. The program also allows for “indeterminate” causes.

2) The “Tariff” method was developed by PHMRC, (Population Health Metrics Research Consortium) [18], and is based on similar considerations: a list of signs and criteria is weighted for each cause using empirical data, the weights are summed for each possible cause, the possible causes are then ranked and the most probable is selected. This method does not allow for indeterminate causes.

3) The “Random Forest” method, also developed by PHMRC [19], is an application of a more general “random forest” procedure, a statistical method of classification based on branching trees. In this case, the classification strategy is based on a learning process from a reference set matching known causes with signs and symptoms, the nodes being based on criteria distinguishing between two causes. The final cause is also based on ranking possible causes. This method allows for multiple causes of death, but seems highly sensitive to any misreporting of a single sign, which may lead to a wrong branch in the tree.

4) The “King-Lu” method was developed by Gary King and Ying Lu [20]. It aims at providing a distribution of deaths by cause from a distribution of signs and symptoms in the population (and not individual causes as for the other methods), through a complex statistical procedure linking causes with signs and symptoms from a reference set.

## Performance of the automated methods

The quality of automated methods can be judged by their statistical performances and, in particular, by comparison with a gold standard, such as a hospital-based medical diagnosis. Several criteria of performance can be used, in particular, sensitivity (proportion of true diagnosis correctly assessed by VAs) and positive predictive value (proportion of VA diagnosis matching the reference set). Specificity and negative predictive values are usually minor issues in this case because of the large number of alternative diagnoses. Articles by Chris Murray *et al*. [16] and by Prabhat Jha and colleagues [12-15] explore the performance of these automated methods. They basically compare the final diagnosis made by verbal autopsy with the hospital diagnosis taken as the reference.

### Sensitivity and positive predictive values

The reports in this collection of papers are not very encouraging with respect to sensitivity and positive predictive value. Automated diagnoses of VAs, even when including additional information from the health system, appear to have abnormally low values for certain diseases assumed to be easy to diagnose: for instance, sensitivity of less than 50% for congenital defects, less than 40% for pneumonia of the new-born, less than 60% for prematurity, less than 40% for children’s diarrhea, less than 50% for children’s pneumonia, less than 50% for adult asthma or epilepsy, and so on.

When properly conducted, physician-based assessments can reach much higher values (for example, >75%) for typical neonatal conditions, infectious and parasitic diseases of children, maternal deaths, external causes and selected non-communicable diseases with typical signs, as shown in the Morocco and Agincourt validation studies [7-10]. In this respect, some of the values reported by Murray *et al*. [16] for physician diagnoses appear abnormally low: sensitivity of 41% for congenital defect, 39% for measles, 38% for children’s pneumonia, 45% for accidental fall, 62% for maternal deaths, 34% for epilepsy, 47% for asthma, and so on, which suggest that the questionnaires used were not optimal.

Therefore, several issues need to be addressed here: whether the problems come from the questionnaire (insufficiently detailed), from the recoding (inappropriate), from the lack of expertise of the physicians, or from the automated procedure. There is obviously room for significant improvements in the future.

### Blind assessment from data mining methods

Another issue of automated diagnoses is the blind assessment in a long list of possible causes from a long series of signs and symptoms using data mining methods. In reality, many causes can be quickly excluded from specific criteria. For instance, if the death is due to a snake bite or to a car accident, there is no need to search for infectious causes: only the timing of death is necessary to make a final assessment. If the death is that of a neonate or is a maternal death, the list of possible causes should be limited to the corresponding causes in the International Classification of Diseases (ICD). These choices could be made with proper recoding of the questionnaire, or using filter questions during the interview.

### The PHMRC Gold Standard

The “PHMRC-gold standard” for verbal autopsies was developed by the Population and Health Metrics Research Consortium, and was made available for public use in October 2013. It is based on a large sample of some 12,000 hospital diagnoses for which verbal autopsies are also available. This sample has a huge potential for testing automated diagnosis procedures. However, it has a number of limitations. First, this is a sample of deaths that occurred in a hospital and for which a diagnosis was made. Therefore, a number of diseases causing death in populations are excluded for a variety of reasons: selection of the populations on which samples are based; exclusion of causes unlikely to lead to death when properly treated in hospital; ignorance of some underlying causes for which only the immediate cause can be identified in the hospital. Second, the final coding of the cause is selective because of the grouping of many causes into an “other cause” category, not counting the fact that some ICD codes used by PHMRC could be misleading (A16, G18, H61 and so on). Third, some typical causes, easy to diagnose by verbal autopsy, are not considered. Fourth, the coding into a grouped cause and two co-morbidity causes (many identical) is not conventional, and differs from the recommended coding procedure into underlying, immediate and contributing cause. As a result, some critical causes of public health importance are not in the final list (cholera, whooping cough, neonatal tetanus, severe malnutrition, kwashiorkor, marasmus and so on). Some causes probably well assessed in the hospital and in the VAs are lumped together in a residual category. With respect to maternal mortality, the distinction between obstetric and non-obstetric causes is difficult, because many seem to be classified under “other pregnancy-related deaths”. Some of these problems are discussed in Peter Byass’s paper [21], in particular the issue of case definition for stillbirths and maternal deaths and the possible confusion between underlying causes and immediate causes.

## Prospects for further work

Much has been learned on verbal autopsies over the past 40 years, but much remains to be improved. Not enough work has been conducted on proper questions to be asked. In particular, the list of questions should not be too short; otherwise, it is difficult to exclude alternative diagnoses. Questionnaires should also be prepared for easy coding and recoding, and allow not only “Yes” and “No” answers, but also “Unknown” or “Missing value”. Computerized questionnaires to be answered in the field with the help of laptop or hand-held (Palm or Personal Digital Assistant) computers could also be developed, with all the necessary filters and branching.

Automated procedures should take into account all the information in the questionnaire and, in particular, should do a first screening on broad categories of causes based on age and sex and on history: neonates, other children, maternal, other adults; communicable, non-communicable, external causes. Then the strategy should be to focus on assessing proper causes within each category.

Most of the available algorithms make little or no use of the duration of the disease. This is, however, crucial information for identifying certain causes, such as cholera, cerebral malaria or stroke.

VAs are not expected to identify all causes in the ICD, which is the standard diagnostic tool for epidemiology, health management and clinical purposes. A category of ‘indeterminate’ is necessary for several reasons: the questionnaire may not be complete or accurate enough to make an assessment; the case may be too complex (several pathologies involved); or the pathology may not be identifiable from interviews. Distributing the indeterminate causes among a list of causes of interest is a separate issue that deserves special care and an imputation strategy.

Some of the procedures mix signs and symptoms with risk factors. These should usually be treated and analyzed separately as risk factors, unless they are critical for the diagnosis (such as working in mines for silicosis).

A cluster of signs and symptoms corresponding to a cause, even when imperfect, and exclusion of other clusters, are probably better pieces of evidence for making a final assessment than scores or branching trees. In this respect, new methods could focus on typical clusters based on critical signs (compulsory or exclusive), and additional signs (adding evidence, optional).

## Conclusions

The work on VAs is important for public health. The Moroccan experience [9,10] is illustrative: the VAs conducted in 1988 quickly revealed that neonatal tetanus was a leading cause of death in the country. When this was presented, the Ministry of Health embarked on a major campaign to eliminate neonatal tetanus, a goal achieved within a few years and certified in 2002.

Even when imperfect, with sensitivity notably lower than 100%, VAs can still be used efficiently for monitoring progress or emerging issues when used in a time series. This simply assumes that the quality of the diagnosis remains constant over time. In this respect standardized questionnaires and automated diagnoses are major assets for ensuring consistency over time. For instance, progress in the control of many infectious diseases (tetanus, measles, whooping cough, cholera, HIV/AIDS, tuberculosis and so on) can be easily monitored by VAs. Progress (or lack thereof) in maternal mortality can also be easily monitored, and information on causes of death is crucial to separate true maternal causes from other pregnancy related deaths [22]. Certain non-communicable diseases can also be easily monitored, such as epilepsy, asthma, kwashiorkor, marasmus, stroke and so on. Some more complex diseases can also be monitored when additional information on medical diagnosis and treatment is available, as is the case for diabetes and hypertension. External causes, such as domestic accident, road traffic accident, snake bites and so on, as well as homicides, deaths from civil unrest and suicides can also be easily captured by VAs.

In the absence of a complete death registration and medical assessment of causes of death, recording VAs on representative samples of deaths in populations remains the only way to obtain this important information.

## Abbreviations

DSS: Demographic Surveillance System; ICD: International Classification of Diseases; PHMRC: Population Health Metrics Research Consortium; VAs: verbal autopsies.

## Competing interests

The author declares that he has no competing interest.

## Authors' information

MG is a demographer with extensive experience in mortality studies in Africa. For some 10 years, he directed the Niakhar DSS (Demographic Surveillance System) in Senegal, contributed to other DSSs in Agincourt, South Africa and Nouna, Burkina Faso, and participated to both studies on verbal autopsies in Morocco. He was instrumental in developing comprehensive questionnaires for verbal autopsies in the early 1980s, and contributed to several scientific meetings on verbal autopsies in the late 1980s and early 1990s.

## References

[B1] Taylor CE Sarma RSS Parker RL Reinke WA Faruqee R Child and Maternal Health Services in Rural India: The Narangwal Nexperiment: Vol. 2 Integrated Family Planning and Health Care 1983 Baltimore: Published for the World Bank by Johns Hopkins University Press

[B2] Grubb GS Fortney JA Saleh S Gadalla S el-Baz A Feldblum P Rogers SM A comparison of two cause-of-death classification systems for deaths among women of reproductive age in Menoufia, Egypt Int J Epidemiol 1988 17 385 391 10.1093/ije/17.2.385 3403135

[B3] Zimicki S Vallin J, D'Souza S, Palloni A Approaches to assessment of the cause structure of mortality: a case-study from Bangladesh Measurement and Analysis of Mortality: New Approaches 1990 New York, NY: Oxford University Press 99 122

[B4] Garenne M Fontaine O Vallin J, D'Souza S, Palloni A Assessing probable causes of deaths using a standardized questionnaire. A study in rural Senegal Measurement and Analysis of Mortality 1990 New York, NY: Oxford University Press 123 142 [reprinted as Public Health Classics, in *Bull World Health Organ* 2006, 84:248–253.]

[B5] Garenne M Fauveau V Potential and limits of verbal autopsies Bull World Health Organ 2006 84 164 10.2471/BLT.05.029124 16583068 PMC2627293

[B6] Kahn K Tollman SM Garenne M Gear JS Who dies from what? Determining cause of death in South Africa’s rural Northeast Trop Med Int Health 1999 4 433 441 10.1046/j.1365-3156.1999.00415.x 10444319

[B7] Kahn K Tollman SM Garenne M Gear JS Validation and application of verbal autopsies Trop Med Int Health 2000 5 824 831 10.1046/j.1365-3156.2000.00638.x 11123832

[B8] Garenne M Kahn K Tollman S Gear J Causes of death in a rural area of South Africa: an international perspective J Trop Pediatr 2000 46 183 190 10.1093/tropej/46.3.183 10893926

[B9] Ministère de la Santé Publique du Maroc Etude Nationale sur les Causes et Circonstances de la Mortalité Infanto-Juvénile (ECCD, 1988–1989) 1989 Rabat, Morocco: Ministère de la Santé Publique

[B10] Ministère de la Santé Publique du Maroc Causes et Circonstances de Décès Infanto-Juvéniles: Enquête Nationale, 1998 2000 Rabat, Morocco: Ministère de la Santé Publique

[B11] Garenne M Darkaoui N Braikat M Azelmat M Changing cause of death profile in Morocco: the impact of child-survival programs J Health Popul Nutr 2007 25 212 220 17985823 PMC2753997

[B12] Aleksandrowicz L Malhotra V Dikshit R Gupta PC Kumar R Sheth J Rathi SK Suraweera W Miasnikof P Jotkar R Sinha D Awasthi S Bhatia P Jha P Performance criteria for verbal autopsy‒based systems to estimate national causes of death: development and application to the Indian Million Death Study BMC Med 2014 12 21 24495287 10.1186/1741-7015-12-21 PMC3912490

[B13] Desai N Aleksandrowicz L Miasnikof P Lu Y Leitao J Byass P Tollman S Mee P Alam D Rathi SK Singh A Kumar R Ram F Jha P Performance of four computer‒coded verbal autopsy methods for cause of death assignment compared with physician coding on 24,000 deaths in low- and middle‒income countries BMC Med 2014 12 20 24495855 10.1186/1741-7015-12-20 PMC3912488

[B14] Jha P Reliable direct measurement of causes of death in low and middle-income countries BMC Med 2014 12 19 24495839 10.1186/1741-7015-12-19 PMC3912491

[B15] Leitao J Desai N Aleksandrowicz L Byass P Miasnikof P Tollman S Alam D Lu Y Rathi SK Singh A Suraweera W Ram F Jha P Comparison of physician-certified verbal autopsy with computer-coded verbal autopsy for cause of death assignment in hospitalized patients in low- and middle-income countries: systematic review BMC Med 2014 12 22 24495312 10.1186/1741-7015-12-22 PMC3912516

[B16] Murray CJL Lozano R Flaxman A Serina P Phillips D Stewart A James SL Vahdatpour A Atkinson C Freeman MK Ohno SL Black R Ali SM Baqui AH Dandona L Dantzer E Darmstadt GL Das V Dhingra U Dutta A Fawzi W Gómez S Hernández B Joshi R Kalter HD Kumar A Kumar V Lucero M Mehta S Neal B Using verbal autopsy to measure causes of death: the comparative performance of existing methods BMC Med 2014 12 5 10.1186/1741-7015-12-5 24405531 PMC3891983

[B17] Byass P Chandramohan D Clark SJ D'Ambruoso L Fottrell E Graham WJ Herbst AJ Hodgson A Hounton S Kahn K Krishnan A Leitao J Odhiambo F Sankoh OA Tollman SM Strengthening standardised interpretation of verbal autopsy data: the new InterVA-4 tool Global Health Action 2012 5 1 8 22944365 10.3402/gha.v5i0.19281 PMC3433652

[B18] James SL Flaxman AD Murray CJ Population Health Metrics Research Consortium (PHMRC) Performance of the Tariff Method: validation of a simple additive algorithm for analysis of verbal autopsies Popul Health Metr 2011 9 31 10.1186/1478-7954-9-31 21816107 PMC3160924

[B19] Flaxman AD Vahdatpoor A Green S James SL Murray CJ Population Health Metrics Research Consortium (PHMRC) Random forests for verbal autopsy analysis: multisite validation study using clinical diagnostic gold standards Popul Health Metr 2011 9 29 10.1186/1478-7954-9-29 21816105 PMC3160922

[B20] King G Lu Y Verbal autopsy methods with multiple causes of death Stat Sci 2008 23 78 91 10.1214/07-STS247

[B21] Byass P Usefulness of the Population Health Metrics Research Consortium gold standard verbal autopsy data for general verbal autopsy methods BMC Med 2014 12 23 24495341 10.1186/1741-7015-12-23 PMC3912496

[B22] Garenne M Kahn K Collinson M Gómez-Olivé FX Tollman S Maternal mortality in rural South Africa: the impact of case definition on levels and trends Int J Womens Health 2013 5 457 463 23950662 10.2147/IJWH.S45983 PMC3741078

